# Genetics and Genomic Medicine in Egypt: steady pace

**DOI:** 10.1002/mgg3.271

**Published:** 2017-01-17

**Authors:** Samia Ali Temtamy, Dalia Farouk Hussen

**Affiliations:** ^1^ Human GeneticsCenter of Excellence for Human GeneticsThe National Research Center (NRC)CairoEgypt; ^2^ Human CytogeneticsCenter of Excellence for Human GeneticsThe National Research Center (NRC)CairoEgypt

## Abstract

Genetics and Genomic Medicine in Egypt: steady pace.

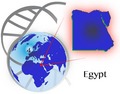

## Geographic and Demographic Data

**Figure 1:**
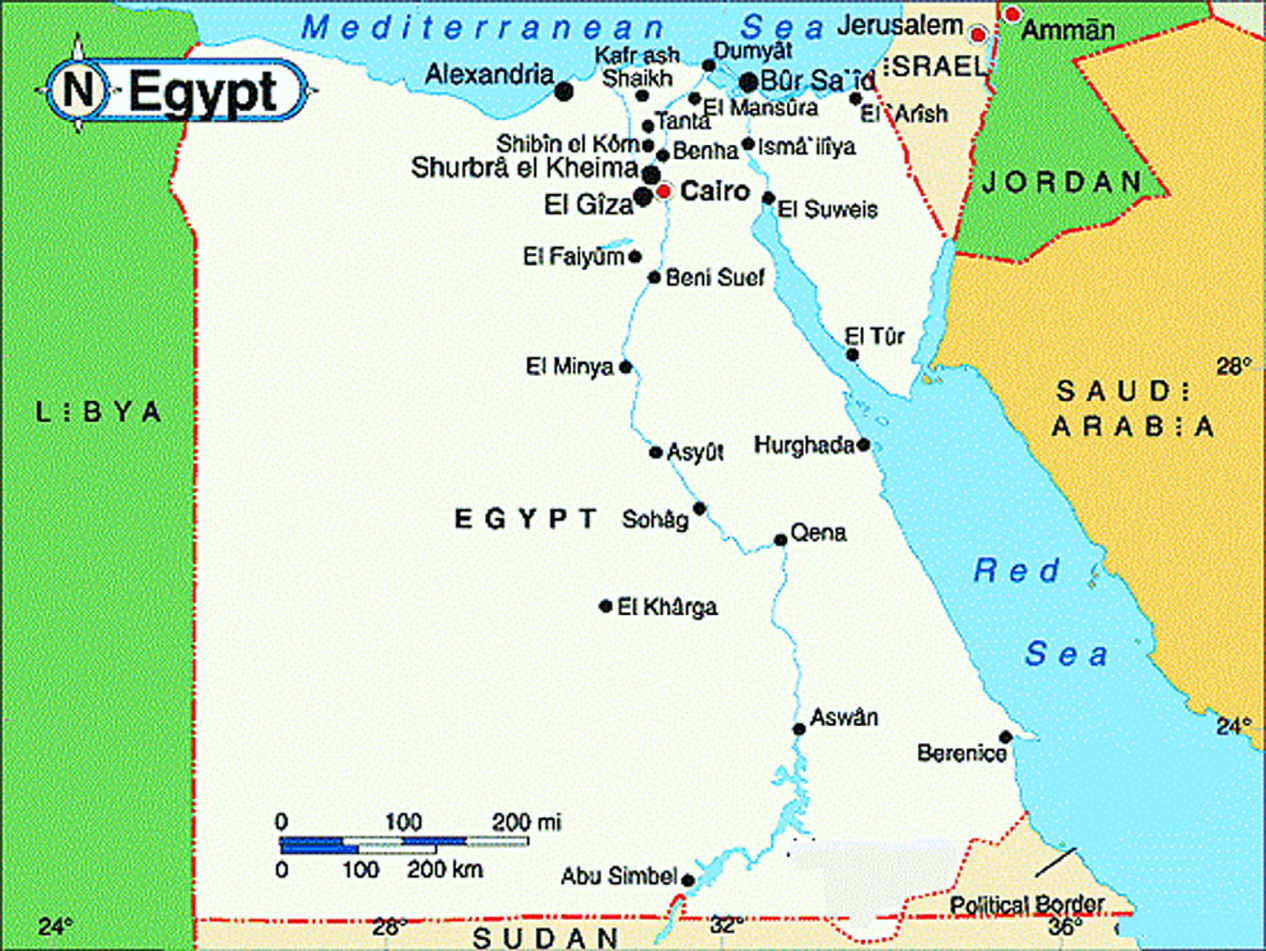
Political map of Egypt.

Egypt is a Mediterranean North African country (Fig. 1). It is the third‐most densely inhabited country on the African continent after Nigeria and Ethiopia. The total number of the Egyptian population is estimated to be 92 million, in 2016 (Central Agency for Public Mobilization and Statistics of Egypt). About 95% of the population live on the sides of the Nile banks, Nile Delta, and along the Suez Canal. Small communities spread throughout the desert regions and clustered around oases.

Both the strategic position of Egypt and its natural resources were attractive to multiple invaders throughout the history. This results in the presence of different genetic origins in the Egyptian population in addition to their Pharaonic origin. Moreover, considerable human movement for migration and trade adds to genetic diversity among the Egyptian population (Temtamy et al. 2010).

## Ancient Egyptians and Genetics

The ancient Egyptian civilization was initiated around 3000 BCE, it was one of the most advanced and productive civilizations throughout ancient history. Evidence of medical organization in ancient Egypt comes from both literature and archeology (Kozma 2006). The dry climate and religious necessity for preservation of dead bodies as mummies (Smith 2000), provided us with a notable indicator of the health status of people during this era (Sullivan 1995).

Ancient Egypt shows some of the earliest evidence for both congenital and acquired diseases. It is a major source of archeological information on achondroplasia and other dwarfing conditions (Kozma 2008).

**Figure 2:**
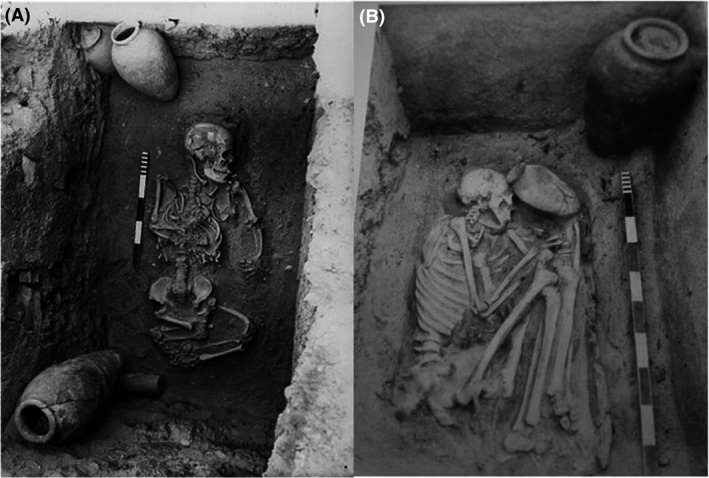
(A) A skeleton of a male achondroplastic dwarf from the Old Kingdom. (B) A skeleton of an average size person from the same burial complex for comparison.

The skeleton of an adult male of normal stature in a tomb of the first dynasty located in Saqqara (a huge ancient necropolis south of Cairo) in the tomb complex of King Wadj is shown adjacent to that of a male with achondroplasia (Fig. 2). It is thought to be from to the period 3100–2800 BCE (Kozma 2010). The long bones are very short and the fibulae bowed. These changes are mostly attributed to short limb dwarfism, most likely achondroplasia (Emery 1954; Weeks 1970).

**Figure 3:**
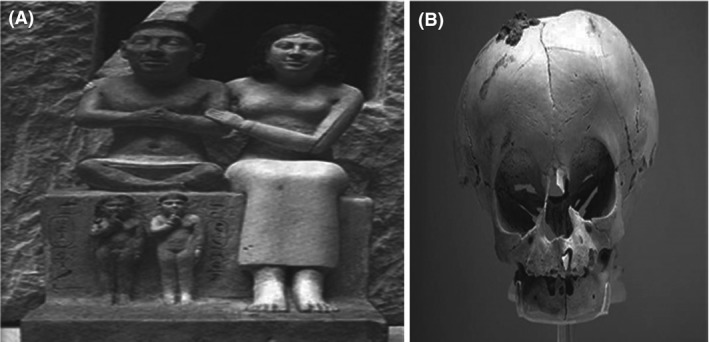
(A) The Statue of Dwarf Seneb with his normal wife and other family members. (B) A skull of a child who suffered from Osteogenesis Imperfecta, 22nd Dynasty. British Museum. Retrieved from http://www.mundieart.com/cabinet.

Another well‐known example is the statue of the dwarf Seneb from the Old Kingdom (4th Dynasty) recording all his family members. Skull evaluation of the statue by Temtamy (1986) suggested the diagnosis of hypochondoplasia. (This statue is included in the Logo of our Center of Excellence for Human Genetics) (Fig. 3A).

An interesting example of a skull, suggested the diagnosis of Osteogenesis imperfecta (El‐Sayed 2008) is demonstrated in a Skull of an Egyptian child of the 22nd dynasty (945–716 BCE) who suffered from osteogenesis imperfecta, now kept at the British Museum, London (Mundie 2008) (Fig. 3B).

Sarry El‐Din (2002) reviewed the documented skeletal abnormalities in ancient Egypt including a probable case of Apert syndrome in a child from Nubia, examples of Klippel‐Feil syndrome dating back to the Ptolemaic period, two cases with transitional vertebrae from the old kingdom and nine cases of achondroplasia in addition to the two previously reported achondroplasia cases from an Egyptian sample related to Giza Old Kingdom (Hussien et al. 1990).

The G→A transition in the FGFR3 gene at cDNA position 1138 diagnostic of achondroplasia was detected in cloned polymerase chain reaction products obtained from the dry mummy of the Semerchet tomb, Egypt (first dynasty, approximately 4890–5050 BP [before present]) by Pusch et al. (2004).

Multiple basal‐cell nevus syndrome (Satinoff and Wells 1969) and alkaptonuria (Stenn et al. 1977) were preserved back in Egyptian history in mummies and skeletons. Two embalmed fetuses were recovered from Tutankhamun's tomb in 1926. One had spina bifida, clubfoot, cleft palate, and hydrocephalus have also been found. Marin et al. (1999) found a band at the level of the HbS mutated fragment in a sample of three predynastic Egyptian mummies, indicating that they were affected by sickle cell anemia.

Temtamy (2005) described the first examples of diastrophic dwarf from ancient Egyptian statues, these statues show short limb dwarfism associated with talipes suggesting diastrophic dysplasia. The burial sites and artistic sources provide glimpses of the positions of dwarfs in daily life in ancient Egypt. Wisdom writings and moral teachings commanded respect for dwarfs and other individuals with disabilities (Kozma 2006).

The Ancient Egyptian civilization ended around 30 BCE, when the Roman Empire conquered Egypt and considered it one of its districts.

## Recent Genetic Facilities in Egypt

With the increasing control of infant mortality due to diarrhea and infectious diseases, genetic diseases are increasingly becoming a health priority. The importance of medical genetics in Egypt started in the Twentieth century in the pediatric departments of Egyptian universities and was well appreciated in the early 1960s at Cairo and Ain Shams Universities by the late Dr Ekram Abdel‐Salam and Dr Nemat Hashem.

**Figure 4:**
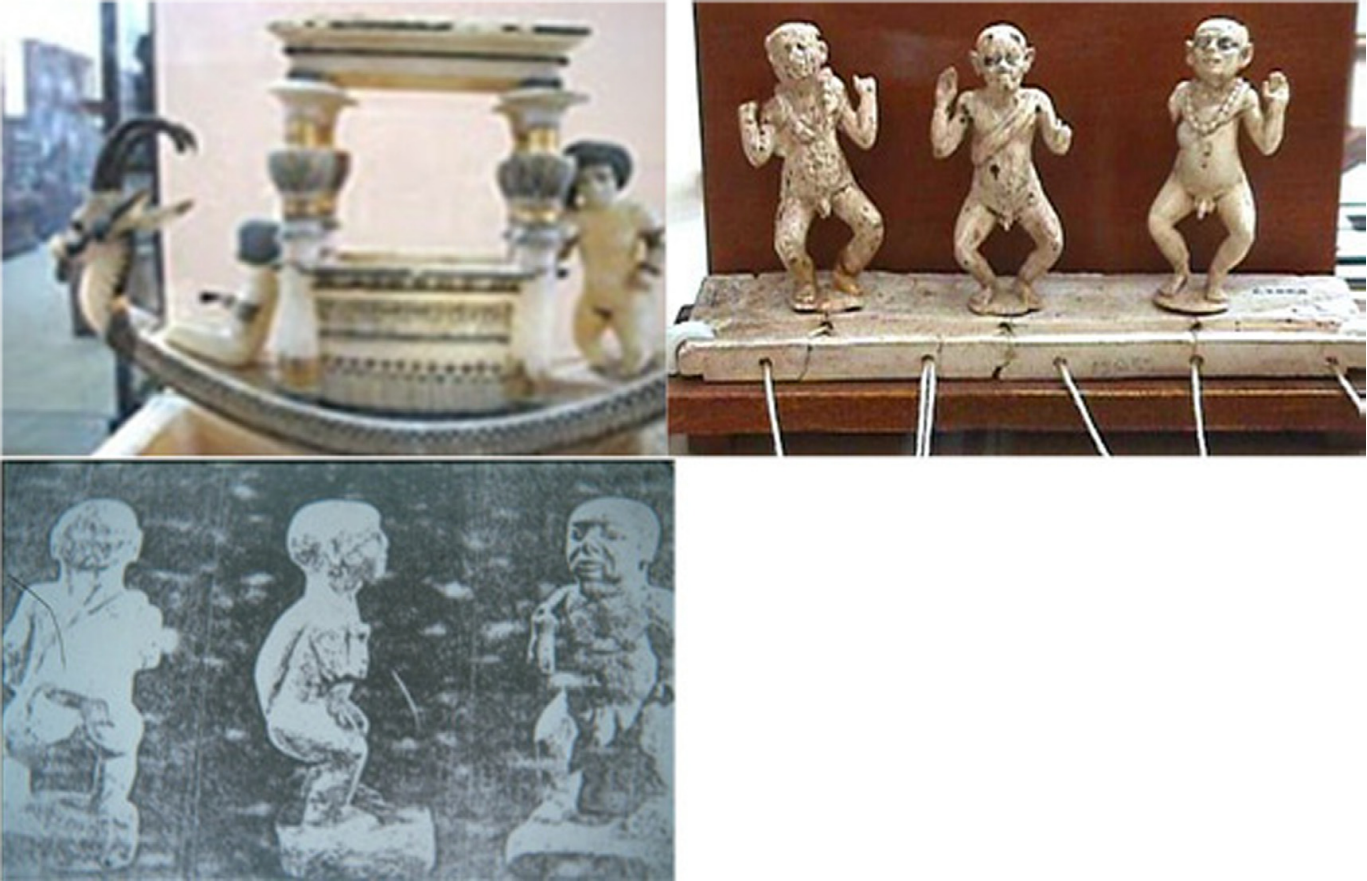
First examples of Diastrophic Dwarfs from Ancient Egypt (Temtamy, 2005; unpublished data).

In 1966, Dr. Samia Temtamy finished her Ph.D. at Johns Hopkins University, USA, in Human Genetics as the first specialized geneticist in Egypt and established the specialty of Human genetics at the National Research Centre (NRC). She published her famous book, The Genetics of Hand Malformations, with Dr. Victor McKusick, in 1978 (Fig. 4) (Temtamy and McKusick 1978).

**Figure 5:**
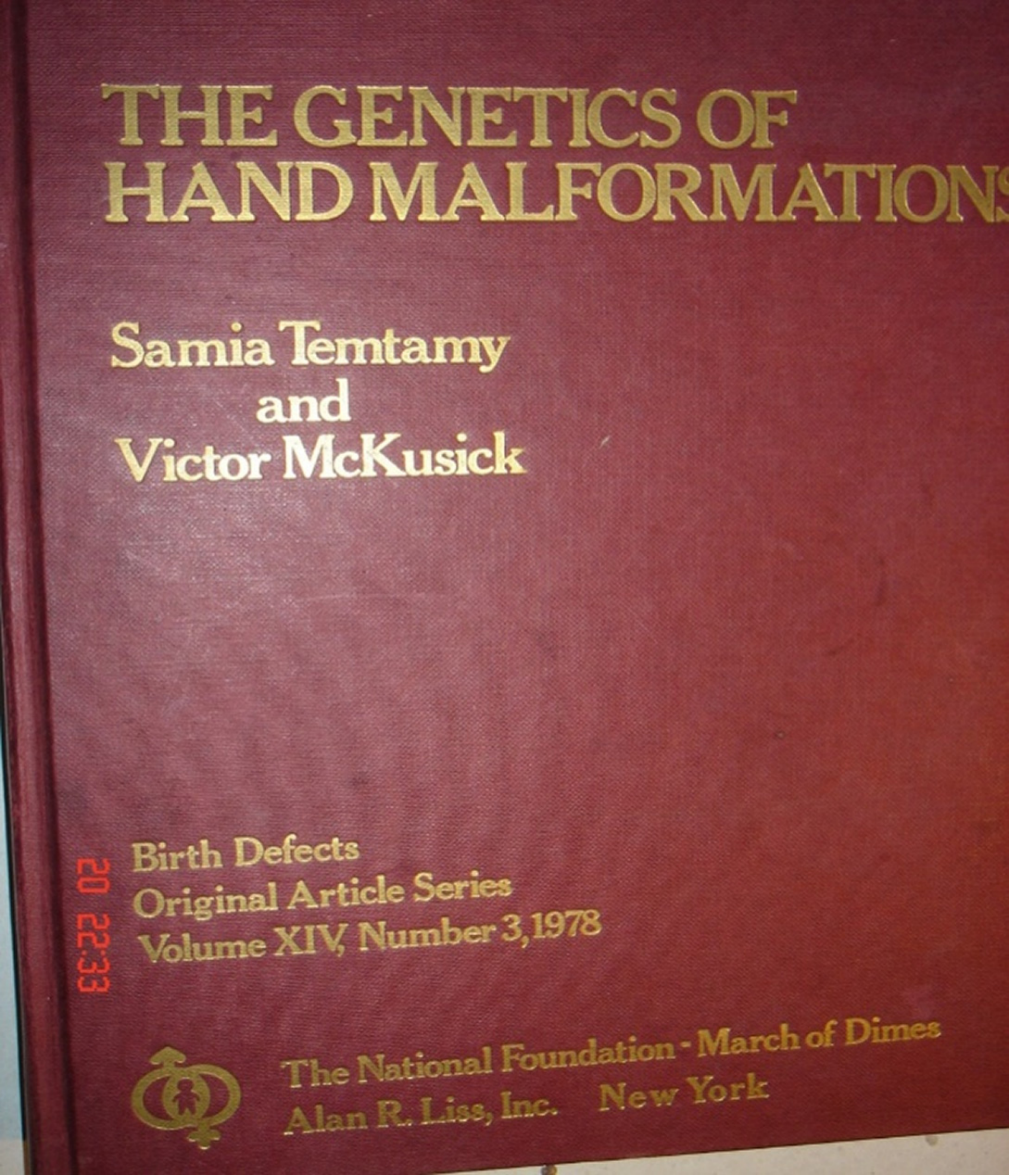
The Genetics of Hand Malformations book (pp. 619).

In 1967, Dr Suzan Roshdy started the medical genetics unit at the Medical Research Institute in Alexandria. This was followed by the initiation of medical genetics units in other universities such as El‐Mansoura and Alexandria Universities. Mubarak City of Scientific Research encompasses centers for frontier sciences including genetic engineering and biotechnology (Temtamy et al. 2010).

After that, Dr. Samia Temtamy established the Human Genetics and Genome Research Division at the National Research Centre until it became the main unit dealing with genetic diseases in Egypt. Today people from all Egyptian governorates and neighboring middle east countries seek diagnosis, treatment, and medical advice at the Center.

Nowadays, human genetic courses are included in the curriculum of medical students in most Egyptian universities. In addition, specialized postgraduate degrees in the field of Medical Human Genetics are offered to graduates from Medical schools in Egypt at Ain‐Shams and Alexandria Universities. Training programs given by specialized geneticists from different institutions including our Division at the NRC are offered to physicians from the Ministry of Health and Population.

## Neonatal Screening Program in Egypt

Neonatal screening for hypothyroidism has been initiated in Egypt as a result of our efforts. In 1991–1995 professor Temtamy was the PI of a research project funded by the Academy of scientific research and technology in collaboration with human genetic experts in the Children Hospital Cairo University, The Medical Research Institute Alexandria University and Pediatric Departments in Ain Shams University and Mansora University. The project was an epidemiologic study on newborns to find out the prevalence of various birth defects including inborn errors of metabolism and hypothyroidism. The results of this project showed a high frequency of Phenylketonuria (1:7000), and of hypothyroidism (1:3000). The results were published in the Journal of the Ministry of Health and Population (Temtamy 1998) to alert policymakers about the need to start neonatal screening for inborn errors of metabolism and hypothyroidism in Egypt. This resulted in the initiation of mass neonatal screening for the 2 disorders.

## Architecture of Human Genetics and Genome Research Division at the National Research Centre

The Human Genetics and Genome Research Division started as a department in 1976, then it developed into a division in 2003, which encompasses a staff of 200 specialists in genetics, distributed in eight departments including: Clinical Genetics, Human Cytogenetics, Medical Molecular Genetics, Molecular Genetics and Enzymology, Biochemical Genetics, Prenatal Diagnosis, Immunogenetics, and Orodental Genetics.

## National Society of Human Genetics‐Egypt

Dr. Samia Temtamy established the National Society of Human Genetics in Egypt in 2005. At the executive committee meeting of the International Federation of Human Genetic Societies (IFHGS), on November 3, 2010, in Washington, DC, USA, The National Society of Human Genetics Egypt was accepted as a corresponding member. Thus, it is the first member in the IFHGS from the Arab world and Egypt.

The Society has multiple targets, the most important are:

1. Increase awareness about human genetics and genetic diseases among the public and professionals to support and participate in research on genetic diseases in Egypt and to attract international attention of specialized scientists.
2. Holding of conferences, workshops, symposia, and training courses in the specialized field of human genetics to increase public and professional knowledge about the different genetic disease and methods for containing them in the society and exchange of scientific knowledge.
3. Encouragement of students and recent graduates to join the activities of the society.
4. Enhancement of national, regional, and international networking and cooperation for early detection, prevention and management of genetic diseases which affect all systems and organs of the human body at the level of Egypt, Arab countries, Africa, Europe, USA, etc.

One of the most important achievements of the society is the publication of the Middle East Journal of Medical Genetics (Lippincott Williams &Wilkins/Wolter‐kluwer) Since Jan 2012.

## Center of Excellence for Human Genetics in Egypt

**Figure 6:**
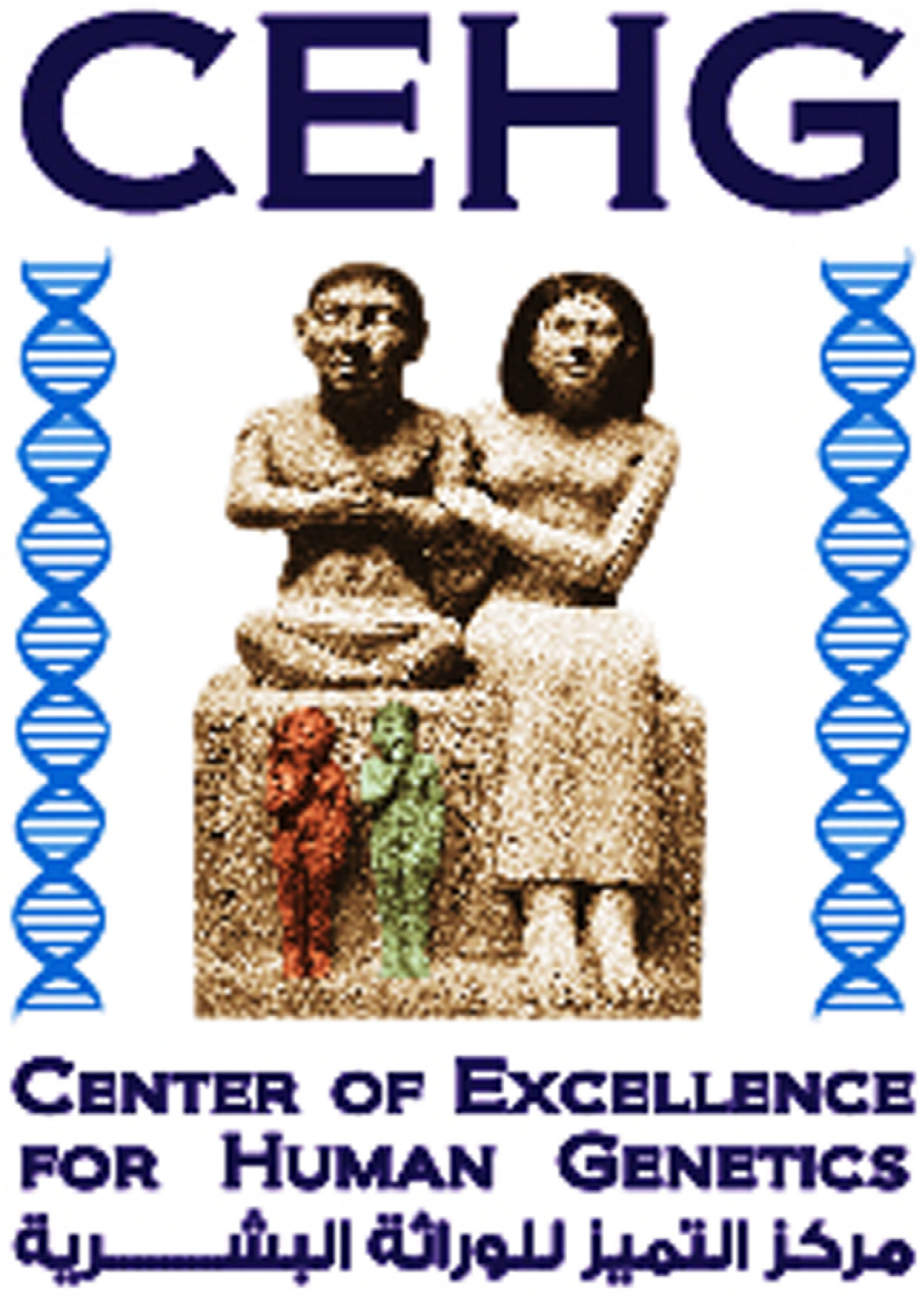
Logo of Center of Excellence for Human Genetics showing dwarf Seneb with his normal wife.

Center of Excellence for Human Genetics (CEHG) at the National Research Center (Fig. 6) has been established in 2014 under the supervision of Dr. Samia Temtamy after implementation of the STDF (Science and Technology Development Fund) project, under the theme: (Advances in the Diagnosis, Management, and Research of Genetic Diseases).

## Genetic Services Provided Through Center of Excellence for Human Genetics

Based on our present capabilities and competence the center provides multiple genetic services through the different genetic departments:

### Multiple specialized human genetic clinics

Multiple specialized human genetic clinics for accurate clinical diagnosis and genetic counseling of different cases including prenatal diagnosis. We also provide enzyme replacement therapy for patients with treatable mucopolysaccharidoses.

One of the main group of disorders studied in detail in our Division are disorders of sex development through clinical, cytogenetic, and molecular investigations. The birth of a child with a Disorders of Sex Development (DSD) is likely to be stressful, especially regarding decisions on gender assignment, and genital abnormalities are associated with stigma and shame. Gender assignment may be biased toward the male gender, because female infertility precludes marriage and female gender adversely affects employment prospects and inheritance. Later gender change in either direction may also carry stigma. Gender reassignment surgery for transsexuals without somatic intersexuality was legalized in Egypt in 2005, but requires permission by a national Sex Identification and Determination Committee (Mazen, 2016).

### Molecular genetic departments for molecular diagnosis of prevalent and rare genetic diseases in four main Categories

#### Neurogenetic disorders

For example, spinal muscular atrophy (SMA), Duchenne muscular dystrophy (DMD), cerebrospinal ataxia, microcephaly, leucoencephalopaties, cerebellopontine hypoplasia, and lissencephaly (Jerber et al. 2016).

#### Limb malformations and skeletal dysplasias

For example, achondroplasia, pycnodysostosis, Winchester syndrome, Dyggve Melchior Claussen syndrome, Temtamy preaxial brachydactyly syndrome, Grebe dysplasia, Pfeiffer syndrome, Roberts syndrome, Robinow syndrome, Osteogenesis imperfecta, hypophosphatasia, and mucopolysacharidoses.

#### Hereditary blood disorders

For example, Thalassemia (*α* and *β*), Thrombosis (Jak 2 gene), G6PD deficiency, Fanconi anemia, and hemophilia.

#### Multiple congenital anomalies

For example, Holt–Oram syndrome, Nager syndrome, RASopathies.

### Human cytogenetics department

Multiple techniques are available for diagnosis of different genetic disease for both prenatal and postnatal diagnosis. These include:

Conventional cytogenetic analysis (CCA), Fluorescent In Situ Hybridization (FISH), Cytokinesis‐block micronucleus assay (CBMN), Multiplex Ligation‐ dependent Probe Amplification (MLPA) analysis, Microarray technique.

### Biochemical genetics department for diagnosis

Biochemical genetics department for diagnosis of mucopolysaccharide disorders, Alpha & Beta mannosidosis disease, Alpha fucosidosis disease, Gaucher disease, Niemann Pick disease, metachromatic leukodystrophy disease, Tay Sachs disease and Sandhoff disease, Cystic Fibrosis and Homocysteinemia. Porphyrias and glycosylation disorders, amino acid, and organic acid disorders using tandem mass analysis. In addition, newborn screening for Phenylketonuria, Biotinidase deficiency, Galactosemia, and Hypothyroidism is offered.

## Genetic Disorders Among Egyptians

Frequent genetic disorders among Egyptians have been summarized by Temtamy et al. (2010).

It is worth emphasizing our study of numerous rare autosomal recessive disorders, for example, Autosomal recessive Robinow syndrome (Aglan et al. 2016) and Roberts syndrome (Ismail et al. 2016), because of the high frequency of consanguinity in Egyptians ranging in various governorates from 27.3% to 46.5% (Temtamy and Aglan 2012) and even delineation of new, previously unknown disorders, for example, Temtamy preaxial brachydactyly syndrome (Temtamy et al. 1998, 2012; Li et al. 2010; Aglan et al. 2014) and homozygous FGFR3 mutation which causes a new autosomal recessive syndrome of tall stature and severe lateral tibial deviation (Makrythanasis et al. 2014); and identification of new genes that cause rare recessive disorders, for example, Ostrix mutation as a cause of autosomal recessive osteogenesis imperfecta in humans (Lapunzina et al. 2010) and *ANTXR1* gene for GAPO syndrome (Hoischen et al. 2013).

Other recent genetic studies in Egypt will be available soon at the National Research Center website; www.nrc.sci.eg.

## Future Prospects

Through the CEHG, provided by an STDF Grant 5253, by the Egyptian government, we are planning and aiming to:

1. Reach accurate diagnosis for different genetic diseases which are a major cause of chronic diseases and crippling in our society.
2. Identify gene mutations prevalent among Egyptians as an essential requirement for their early diagnosis, prevention, and management.
3. Study of rare genetic diseases may lead to the discovery of new diseases or genes and genetic pathways thus paving the way for novel therapeutic or preventive strategies.
4. Acquisition of modern equipments will permit training our researchers on the new and emerging diagnostic molecular technologies.
5. National and international collaboration to allow mutual exchange of expertise and twinning agreements between related centers of excellence worldwide.

## Conflict of Interest

None declared.
